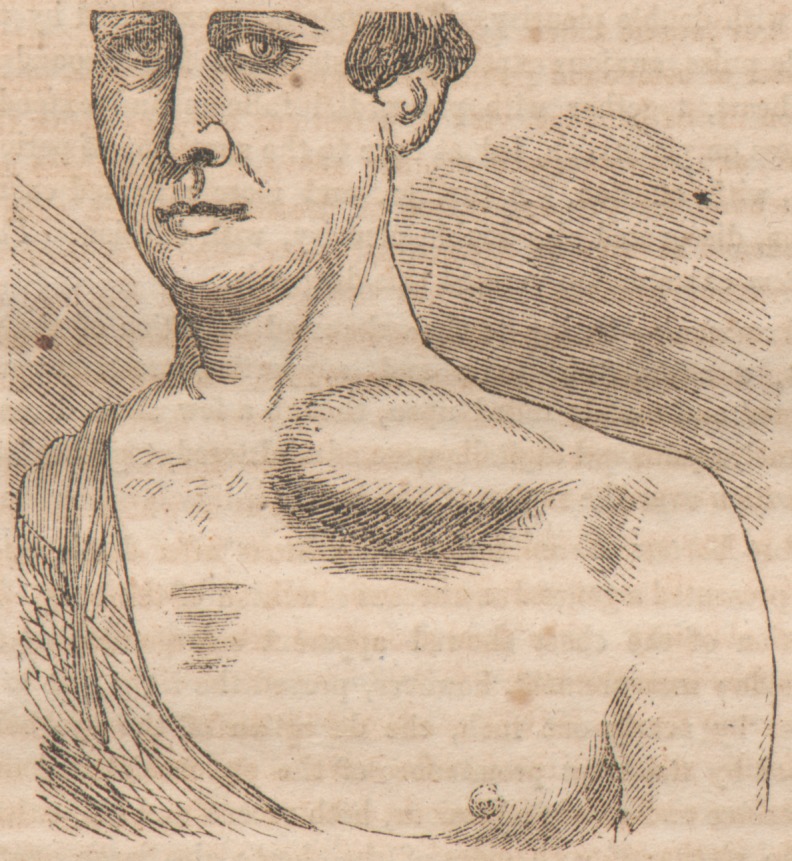# Resection of Three-Fourths of Clavicle for Osteo-Sarcoma

**Published:** 1864-05

**Authors:** Charles Bell Gibson

**Affiliations:** Richmond.


ART. IV.—*Resection of three-fourths of Clavicle for Osteo-Sarcoma.* By Surg. CHARLES BELL GIBSON, Richmond.

Jackson Neill, farmer, Scott county, Virginia, age 40, general health good, was brought to me by Dr. Carmack of same county, for removal of an osteo-sarcomatous tumor of left clavicle.

It appears that some twelve months ago Mr. Neill had a fall which partially fractured the clavicle, and that eight months subsequently he received a blow, from the sudden and violent starting of a horse, against the shoulder of the same side, which, it was supposed, again fractured the bone A sudden enlargement of the bone occurred immediately after this injury, which subsided in a few days, under diligent use of cold water, leaving, however, a small tumor near the sternal articulation.

This tumor increased in size steadily, extending along the shaft of the clavicle towards the acromial end, downwards on the chest, and upwards and backwards into the neck. It is at this time larger than his fist, and has the mixed hard and soft feel of osteo-sarcomatous tumors. It extends from the sternal articulation three-fourths the length of the clavicle, tapering down to the point from its extreme height, which is about an inch exterior to the sternal articulation.

There is no pain in the tumor, and the only inconvenience is slight difficulty of deglutition if patient is lying on the left side. His respiration is not at all affected.

Pulsation of carotid and axillary arteries distinct. Subclavian artery not felt pulsating.

Jan 30th.—*Operation:* Present Surgeons Peticolas, Sorell, Michel, J. B. Read, J. S. D. Cullen and H. L. Thomas, and Dr. Carmack.

An incision was made half an inch interior to the sternal articulation, over the middle of the tumor and along the middle of the clavicle to its outer fourth portion. The integument was dissected upwards and downwards from the tumor and bone, and the attachment of the pectoralis major and first part of deltoid muscles divided. The upper and lower borders of the clavicle being freed and fully exposed, and the subclavian muscle separated by the knife-handle, a large blunt curved needle was passed along the under surface of the clavicle from before backwards, carrying with it a chain-saw, and the bone sawn through at the commencement of its outer fourth portion.

The end of the bone was now seized by Fergusson's "lion forceps" and raised, and the attachments of the subclavin muscle very carefully divided.

The sternal articulation was opened in front with caution, and the tumor became easily moveable. The dissection was continued to its posterior aspect, and its connections gradually detached. The sterno-cleido-mastoid muscle afforded an anterior and posterior investment to the tumor, its fibres being spread out in the form of a fan, and completely separating the tumor from the vessels and nerves of the neck. With great care the tumor was gradually dissected from the posterior investment of the sterno-cleido-mastoid muscle, and the disarticulation being completed from behind, at the sternal articulation, the tumor was finally raised and entirely removed from its bed.

Two small muscular arteries required ligature, and a branch of the external jugular vein was also tied. The amount of blood lost did not probably exceed four ounces, and the time occupied in removing the diseased mass was about forty minutes.

The line of incision was connected by silver wire sutures, a thick and soft compress applied over the flap and sustained by a bandage which also secured the arm to the side of the chest and supported the elbow.

The formidable description of Dr. Mott's *"Waterloo Operation,"* as he calls it, in which forty ligatures were applied, and four hours required for its performance, had not prepared the operator for the facility with which this large tumor was removed, nor for the entire concealment of important arteries, veins and nerves. So complete and protecting, in fact, was the muscular septum between them and the tumor (the expanded sterno-cleido-mastoid) that even the pulsations of the two great arteries were not visible. The subclavian vein, always an object of great anxiety in this operation, was not seen at any time in the dissection along the under surface of the clavicle or tumor.

Jan. 30th, 6 o'clock P. M.—Patient comfortable. Ordered tincture opium forty drops.

Jan. 31st, 10 A. M.—Patient has slept tolerably well; has had his breakfast; suffers no pain; is very comfortable.

Feb. 1st, 10 A. M.—A good night. Appetite good. Ordered sulphate magnesia half an ounce.

Feb. 2d.—Dressed wound. Union at inner and outer portions to the extent of three inches each way.

Feb. 2d, 3d, 4th, 5th, 6th and 7th.—Visits and dressing each day. A little brandy and water administered three times each day, from regard to old habits. Appetite and sleep perfect. Patient takes exercise about his room. Flap adherent to tissues beneath. Discharge healthy, not abundant. Granulations of central portion of line of incision a little pale, but improving.

Feb. 9th, 10th, 11th.—Daily visits and dressings.

Feb. 21st.—Wound entirely closed, and patient leaves for home to-morrow. Arm sustained by sling and kept closely to side of chest.

## Figures and Tables

**Figure f1:**